# Reproductive planning in times of Zika: getting pregnant or delaying plans? The opinion of the Brazilian Society of Assisted Reproduction Committee - a basis for a bioethical discussion

**DOI:** 10.5935/1518-0557.20160034

**Published:** 2016

**Authors:** Bruno R. de Carvalho, Paulo F. Taitson, Karina S. A. G. Brandão, Rui Alberto Ferriani, Hitomi M. Nakagawa, Adelino A. Silva, Joaquim R. C. Lopes, SBRA - Brazilian Society of Assisted Reproduction Committee

**Affiliations:** 1 GENESIS - Center for Assistance in Human Reproduction, Brasília, DF, Brazil; 2 Pontifical Catholic University of Minas Gerais, Belo Horizonte, MG, Brazil,; 3 CENAFERT, Salvador, BA, Brazil; 4 School of Medicine of Ribeirão Preto, University of São Paulo, Ribeirão Preto, SP, Brazil; 5 President of SBRA - Brazilian Society of Assisted Reproduction

**Keywords:** Zika, microcephaly, human reproduction, bioethics, central nervous system disorders

## Abstract

Although the causality between Zika virus, microcephaly, and other central nervous system disorders has been taken for granted by the scientific community, many uncertainties remain. The gap of knowledge at the moment is large enough to remove part of the confidence physicians have on the advice given to patients - and infertile women in particular - on their reproductive plans. Pretreatment serologic screening is a possible strategy to offer more confidence for individuals choosing to bear children regardless of the Zika virus, but the tests currently available do not seem to be sufficiently adequate. Until now, there is no formal recommendation to avoid pregnancy solely because of the Zika virus outbreak, and the choice of becoming pregnant has been regarded as a personal decision to be made by each woman and her family.

## Zika virus

In the middle of the last century, child bearing was considered a natural phenomenon, the outcome of the reproductive union of a couple. Conception was seen as something final, and did not arouse greater speculations or questions from society or science. The desire to have children is an innate sense, inherent to the protection and survival of all species, but with the advent of the Zika virus (ZIKV), women have been advised to postpone pregnancy. The advocates of such advice find support in the association between ZIKV, microcephaly, and other disorders of the central nervous system (CNS), and in the observation of other adverse events with the offspring.

ZIKV is a flavivirus closely related to the dengue, West Nile, Japanese encephalitis and yellow fever viruses. In humans, it causes a disease known as the Zika fever. The virus was first isolated in 1947 from the serum of a Rhesus monkey in the Zika forest in Uganda; the virus was isolated in humans in 1954 in Nigeria. Evidences of human infection have been reported in other African countries such as Uganda, Tanzania, Egypt, Central African Republic, Sierra Leone, and Gabon, and in parts of Asia including India, Malaysia, the Philippines, Thailand, Vietnam and Indonesia from 1951 to 1981 (World Health Organization, 2015).

The disease is transmitted by the Aedes aegypti and other Aedes mosquito species, such as Aedes africanus, Aedes apicoargenteus, Aedes furcifer, Aedes luteocephalus and Aedes vitattus. In 2009, it was suggested that ZIKV could also be sexually transmitted between humans. Professor Brian Foy, a biologist of the Arthropod-borne and Infectious Disease Laboratory at Colorado State University, visited Senegal and was bitten on several occasions during his research. A few days after returning to the United States, Foy showed symptoms of ZIKV fever, but not before having sex with his wife, who later developed symptoms of the disease. Foy was the first person known to have transmitted the virus to another human being by sexual contact (Taitson, 2016; Campos *et al*., 2015).

ZIKV infection was first detected in Northeastern Brazil in early 2015, in several patients presenting mild fever, rash, conjunctivitis and arthralgia. On April 29, 2015, researchers at the Federal University of Bahia (UFBA) reported the identification of ZIKV by reverse transcription polymerase chain reaction (RT-PCR) in eight of 25 samples collected in the region of the city of Camaçari (MICROCEFALIA - Ministério da Saúde divulga boletim epidemiológico - Brazil, 2015). On May 9, 2015, the Oswaldo Cruz foundation (Fiocruz) identified ZIKV by the same technique in eight of 21 samples collected in the city of Natal, in the state of Rio Grande do Norte (Zanluca *et al.*, 2015). Since then, almost all Brazilian states have identified the circulation of suspected cases of ZIKV fever; the autochthonous transmission of ZIKV has been confirmed in 38 countries and/or territories in the Americas. According to official Epidemiologic Report number 25 (ER25) from the Public Health Emergency Operations Center on Microcephaly, there had been ten confirmed cases of sexually transmitted ZIKV infection until May 5 in five countries: Argentina (1), Canada (1), Chile (1), Peru (1) and United States (6) (Centro de Operações de Emergências em Saúde Pública sobre Microcefalias - Brazil, 2016).

## Brazilian outbreak

Brazil has faced a ZIKV outbreak since mid 2015. Given that 80% of the infected individuals do not show signs or symptoms of disease and most of the patients do not seek treatment at a health care center, it is impossible to know the actual number of cases of infection by ZIKV. The use of RT-PCR, the best ZIKV detection method, is limited to the early acute stages of infection (≤ 7 days) and serological tests have been only recently available. Considering these diagnostic limitations, the number of ZIKV cases was estimated from the number of patients ruled out for dengue and projections based on the international literature. Thus, the estimated number of ZIKV infections in Brazil since the beginning of the outbreak varies between 872,347 to 2,734,911 cases, considering only the States with ZIKV autochthonous circulation confirmed by a reference laboratory. According to the Ministry of Health, Brazilian research institutes are in the process of producing more accurate projections (MICROCEFALIA - Ministério da Saúde divulga boletim epidemiológico, Brazil, 2015).

The number of cases of microcephaly reported in Northeastern Brazil increased dramatically since October of 2015 (Kleber de Oliveira *et al*., 2016). According to the ER25, 7,438 cases of microcephaly and/or other disorders of the CNS in newborns, stillbirths, miscarriages or fetuses were notified in Brazil between November 8, 2015 and May 7, 2016. This number includes the previous definition of operational case - normal head circumference ≥ 33 cm - and the criteria for microcephaly adopted by the surveillance protocol from December 09, 2015, which defined a minimum accepted head circumference of 32 cm for full term newborns. The reported cases were distributed among 1,394 cities, but 5,706 cases (76.7%) were concentrated in the Northeast region. Most suspected cases (n = 1,930), accounting for 25.9% of the total number of cases registered across the country, are in the state of Pernambuco, the first to identify an increase in the number of cases of microcephaly (Centro de Operações de Emergências em Saúde Pública sobre Microcefalias- Brazil, 2016).

The ER25 accounted for 4,004 completely investigated cases, and the existence of microcephaly and/or other CNS disorders suggestive of congenital infection was confirmed in 1,326 cases (33.1%). However, only 205 (15.5%) of the cases with a confirmed association had ZIKV identified by means of laboratory tests (PCR and/or serology); the rest was diagnosed based on clinical and/or radiological criteria: typical changes indicative of congenital infection, such as intracranial calcifications, dilation of cerebral ventricles or changes in the posterior fossa, and other clinical signs observed by imaging (Centro de Operações de Emergências em Saúde Pública sobre Microcefalias - Brazil, 2016).

## ZIKV, microcephaly, and other central nervous system disorders

Although the Brazilian Ministry of Health took the association between ZIKV and microcephaly for granted in early 2016, the scientific community seemed to be divided on the subject until a few weeks ago. Despite the identification of ZIKV in blood and tissues of fetuses and infants with microcephaly or other CNS disorders, the vast majority of the clinical data were obtained retrospectively, and many of the clinical and radiological findings were nonspecific, requiring careful differential diagnosis against other infectious diseases.

ZIKV was found in the amniotic fluid (Calvet *et al*., 2016) and in the tissues of miscarried fetuses and newborns dead shortly after birth (Martines *et al*., 2016). In all cases described by Martines *et al*, the mothers had presented clinical signs of ZIKV infection during the first trimester of pregnancy. Researchers found significant changes in histopathology parameters in the brains of newborns, such as calcified parenchyma, microglial nodules, gliosis, cell degeneration, and necrosis. One of the miscarried babies had heterogeneous chorionic villi calcifications, fibrosis, fibrin deposition between villi and focal villitis (Martines *et al.*, 2016). A connection between ZIKV and other disorders of the central nervous system (CNS), fetal hydrops or fetal death has been described in the literature (Sarno *et al*., 2016; Microcephaly Epidemic Research Group, 2016).

In spite of the virus' proven ability to cross the placenta and affect a developing fetus, until recently there was no consensus over the quality of the scientific evidence to establish a causal link between CNS anomalies and ZIKV (Faria *et al*., 2016; Tetro, 2016). On April 13, Rasmussen *et al* suggested that the literature now provides sufficient evidence to establish a causal relationship between prenatal ZIKV infection and microcephaly or other serious CNS anomalies, based on specific criteria for the evaluation of potential teratogens (Shepard's criteria) and criteria for causation (Bradford Hill's criteria) (Rasmussen *et al*., 2016). Since then studies with pregnant mice (Miner *et al*., 2016; Lazear *et al*., 2016) have looked into the effects of infection by ZIKV and provided significant information on vertical transmission and ZIKV-related pathogenesis, reinforcing the causal role of the virus in neurological anomalies observed in humans.

## The outbreaks in Brazil, Colombia and French Polynesia

A recent Brazilian study assessed 88 women at five to 38 weeks of gestation from September 2015 to February 2016; seventy-two of them (82%) were positive for ZIKV in blood and/or urine tests. Forty-two ZIKV-positive women (58%) underwent ultrasound examination, and 12 cases of fetal abnormalities were detected. The adverse outcomes reported in the study included two intrauterine deaths at 36 and 38 weeks of gestation; five cases of intrauterine growth restriction with/without microcephaly; seven cases of CNS injury, especially ventricular calcifications; seven fetuses with abnormal changes in amniotic fluid volume or flow changes in the brain or umbilical arteries (Sikka *et al*., 2016).

Many consider the data on ZIKV and CNS anomalies obtained to date sufficient, but more evidence is required for the causal relationship to gain strength. At least two situations call for further elucidation. The first occurred in Sergipe, the Brazilian State with the largest number of cases of microcephaly per population, where only recently cases of infection by ZIKV have been identified. Among the cases of microcephaly in Sergipe, samples of pregnant women and children from the city of Itabaiana were analyzed with the aid of researchers from the University of São Paulo; ZIKV antibodies were found in 7/8 women and 4/8 children. Although these preliminary results confirm the circulation of ZIKV in the State, another 172 blood samples were found to be negative for ZIKV, and 976 samples were still awaiting assessment until March 19 (Secretaria de Estado da Saúde - SES - Sergipe, 2016).

The other situation concerns the numbers from Colombia. Since the confirmation of ZIKV circulation in the country and the beginning of the epidemic phase in mid-2015 to mid-March 2016, there have been 2,355 laboratory-confirmed cases, 46,556 cases confirmed by clinical criteria, and 6,813 suspected cases (Instituto Nacional de Salud - Colombia, 1016). According to the last issue of the World Health Organization Situation Report, the Colombian outbreak seems to be in decline, as no additional cases of microcephaly have been reported in the country (World Health Organization, 2016a).

In the specific case of Brazil, one of the critical issues to be addressed refers to the actual incidence of microcephaly in the country. The increase observed in cases of microcephaly might be largely attributed to the intense search for malformations encouraged by media reports and the strong suspicion of their association with ZIKV, in addition to misdiagnoses of pre-Zika phase disease, since there is no consensus over diagnostic criteria (Butler, 2016).

In 2010, the Live Births Registry (SINASC) of the Brazilian Ministry of Health described an incidence of microcephaly of 5.7/100,000 live births, a very similar ratio to what had been described ten years before, which would correspond to 176 neonates born with the malformation (Simmins Jr, 2016). However, a recent study held in the State of Paraíba and published by the World Health Organization discussed the underreporting of microcephaly cases in the country before the ZIKV outbreak. In the Paraíba study, the data from 16,208 children born in public hospitals between January 2012 and December 2015 revealed a prevalence of congenital microcephaly between 4% and 8%, depending on the criteria used. If these numbers were compared to the total number of live births in Paraíba in 2014 (n = 58,147), 4,652 cases of microcephaly would have occurred in the State according to the criteria adopted by the Brazilian Ministry of Health; Fenton growth charts would yield a total of 2,442 cases; 2,907 cases would have occurred according to proportionality criteria; or yet 1,105 cases would have been found if all diagnostic criteria were considered together (Soares de Araújo *et al*., 2016). And if they were applied to the country as a whole, those percentages would yield an incidence of 1,900/100,000 live births per year. In other words, the estimated number of cases of microcephaly each year in Brazil would be greater than 56,000 (Simmins Jr, 2016).

A retrospective study by Cauchemez and colleagues looked into the ZIKV outbreak occurred in French Polynesia between October 2013 and April 2014, and found a prevalence of microcephaly of two cases per 10,000 newborns, and a risk of microcephaly associated with ZIKV of 95 cases per 10,000 women infected in the first trimester, or 1% (Cauchemez *et al*., 2016). Researchers analyzed viruses circulating in Brazil and other countries in the Americas (Martinique, Colombia, Haiti, Guatemala, Suriname, Puerto Rico) and Asia (French Polynesia, New Caledonia, Cook Islands, Easter Island, Vanuatu, and Solomon Islands), and concluded that the strain closest to the one emerged in Brazil comes from French Polynesia (Musso, 2015). This confirmation may allow Brazilian authorities to assume the same level of risk until more reliable local data is available.

New statistical data have been published in a recent study, but the authors admitted that the level of uncertainty is still significant and that such a limitation might be associated with unknown infection rates, especially in recently exposed populations. According to Johansson *et al.* (2016), microcephaly rates could vary from 1% to 13% depending on the percentage of the population infected with the virus. Assuming that 10% of the population of Bahia had been infected, the authors estimated the prevalence of microcephaly at around 13% secondary to infection in the first trimester; however, if 80% of the population in Bahia contracted ZIKV, the risk would be close to the levels indicated by the study carried out in French Polynesia.

In the face of uncertainty and based on the recent recommendations of the World Health Organization on microcephaly and its relation to ZIKV (March 9, 2016), the Brazilian Ministry of Health adopted new parameters to measure the head circumference of newborns and identify suspected cases of microcephaly. Full term male and female infants are expected to have head circumferences greater than 31.9 cm and 31.5 cm to be categorized as normal, respectively. For preterm infants, the new recommendation replaced the diagnostic parameters of the Fenton growth curves with the guidelines established by the International Fetal and Newborn Growth Consortium for the 21st Century, known as Intergrowth (Ministério da Saúde - Brazil, 2016).

## The decision to conceive

Some questions remain unanswered. Is it right to counsel women to postpone pregnancy plans? If so, when would it be safe to get pregnant? How can the risk of having ZIKV-related microcephaly within the first months of gestation be predicted? What advice should be given to them if the numbers become more disappointing in the future?

Specialists in infectious diseases agree that the recent increase in the number of cases may be due to the absence of immunity in much of the population in countries where outbreaks have been reported, but they differ when it comes to forecasting the future behavior of ZIKV. In one scenario, it has been assumed that ZIKV may repeat the behavior of other arboviruses such as dengue, with regular recurrences and small peaks of cases during the rainy season. On the other hand, ZIKV may disappear in a few years and spend decades in silence (Branswell, 2016). This uncertainty prevents health care workers from recommending the postponement of pregnancy for a specific period of time. No one can tell with certainty how long a woman should wait before getting pregnant.

The gap of knowledge at the moment is large enough to remove part of the confidence physicians have on the advice given to patients - and infertile or women of advanced reproductive age in particular - on their reproductive plans. It may be prudent to postpone pregnancy in ZIKV-affected regions. Some health authorities consider the circumstantial evidence available too strong for anyone to take their chances. However, many believe that as long as they are provided with good information, women should decide whether they want to get pregnant or not.

The Pernambuco Health Department stated that every woman should be advised individually about ZIKV and microcephaly. And a health care provider they trust should lead the counseling process. According to the institution's clinical and epidemiologic research protocol for microcephaly, there is no formal recommendation to avoid pregnancy, and the decision of getting pregnant is a matter of personal decision for each woman and her family (Secretaria Estadual de Saúde de Pernambuco, 2015). The representation of the Pan American Health Organization/World Health Organization (2016) in Brazil adopts the same position.

It may be precocious and excessively invasive to advise women to postpone their plans of pregnancy, for the simple fact that no one can affirm that an outbreak of microcephaly is in effect. Formal records are under suspicion and misdiagnosed cases of microcephaly in Brazil might be subject to an independent investigation. In fact, the scientific information to date only points to the emergence of a new microcephaly causative agent, as a result of vertical transmission. And this may be the sole conclusion in the end of the story. Assuming the causation mechanism has been correctly identified, ZIKV should be added to the TORCH complex as a new "other" agent. It has been suggested that researchers may look at diseases like rubella as potential models for how ZIKV damages the fetal CNS and how outbreaks can be stopped (Lafrance, 2016).

Caution is required and panic must be avoided. The population must be advised to eliminate the mosquito and prevent mosquito bites, since these are the two most important protective actions available at the moment. Special attention is required from people at risk and pregnant women in particular (World Health Organization, 2016b). Patients and partners must be encouraged to use repellants, window nets, and protective screens, put on long-sleeved shirts and long pants, and wear condoms while having sex without reproductive purposes.

There is no consensus on how to counsel women - infertile women and individuals of advanced reproductive age in particular - on whether they should postpone their plans of getting pregnant. Women opting to wait until the matter has been resolved may choose to have their oocytes cryopreserved, thus providing them with a chance of enjoying biological motherhood at a later time in their lives.

## ZIKV screening

The use of pre-pregnancy serologic screening is a possible strategy to offer more confidence for those who prefer to carry on with their plans of conceiving despite the ZIKV outbreak. However, the high cost of screening has hampered the introduction of these tests in public and supplementary health care services (Sociedade Brasileira de Patologia Clínica/Medicina Laboratorial, 2016).

Laboratory diagnosis of ZIKV may be achieved directly by RT-PCR analysis, which allows the detection of the virus itself. The molecular test can detect the presence of ZIKV in blood within the first seven days of exposure; in urine samples, PCR can identify ZIKV for a period of 15 days since the time of infection. Negative blood or urine RT-PCR tests cannot rule out infection if the contact with the virus occurred seven to 15 days before the samples were collected. Suspected cases require antibody testing (Sociedade Brasileira de Patologia Clínica/Medicina Laboratorial, 2016).

RT-PCR is effective only during the very early acute phase of infection. Therefore, serological tests appear to be an option in ZIKV screening. Indirect immunofluorescence, immunochromatography, enzyme-linked immunosorbent assay (ELISA), and plaque-reduction neutralization test (PRNT) are currently available for ZIKV identification. In indirect testing methods, the presence of IgM antibodies characterizes acute infection; IgM is detectable four days after exposure and remains constant for up to 12 weeks. In theory, a negative serological test after 12 weeks from the supposed exposure rules out infection (Sociedade Brasileira de Patologia Clínica/Medicina Laboratorial, 2016).

According to the Brazilian Society of Clinical Pathology and Laboratorial Medicine, the sensitivity and specificity of the ZIKV serology kits registered with the Brazilian National Health Surveillance Agency (Anvisa) range from 96.8% to 100%, and 96.6% to 100%, respectively. However, the accuracy and applicability of the tests have been questioned because of the number of false positive results caused by cross-reactions with other viruses (Sociedade Brasileira de Patologia Clínica/Medicina Laboratorial, 2016). Comparative neutralization tests may provide higher specificity. PRNT may produce four-fold increases in neutralizing antibody titers in the absence of increases in antibody titers by other flaviviruses, which is considered sufficient evidence of recent ZIKV infection (World Health Organization, 2016c).

## Regulations for assisted reproduction

Facing a ZIKV outbreak and the possibility of a microcephaly outbreak, the regulatory board at Anvisa revised the regulations for the operation of cell and germ tissue banks (CGTB). Since March 30, women undergoing ovulation induction for in vitro fertilization or oocyte cryopreservation procedures and biological material donors in Brazil must be tested for ZIKV before any material is collected. The aim of the new rule is to avoid contamination by ZIKV of children conceived through assisted reproduction technologies, given the possibility of the disease being transmitted sexually (ANVISA, 2016).

According to the document published by Anvisa, CGTBs can only collect gametes or germ tissue for use in assisted reproduction procedures after obtaining non-reactive or negative test results for ZIKV infection no more than five days prior to gamete collection; individuals whose laboratory tests yield positive or inconclusive results will be temporarily suspended from treatment and tested again 30 days later (ANVISA, 2016).

A major challenge for many reproductive medicine centers is the five-day period for gamete collection, which in practice only enables ZIKV testing by PCR, once IgM testing may take up to eight days and the rapid test is not broadly available. The official document clearly establishes IgM as the standard test; formally, PCR, which is faster, is not included in the standard, but it should be seen as an option when the patient does not wish to postpone treatment.

## Remaining questions

Assuming there is a causal relationship between ZIKV and adverse birth outcomes, researchers are expected to devote their efforts to shed light on the many still unclear points and minimize the virus burden. Understanding serologic curves in bodily fluids and the full spectrum of phenotypes in congenital ZIKV infection syndrome is definitely a target, as is the quantification of relative and absolute risks among exposed fetuses in different times during pregnancy and the factors impacting risk levels (Rasmussen *et al*., 2016).

## Summary

- Many aspects related to the transmission of ZIKV and its effects on human health are not fully understood by researchers; educational initiatives must be devised to reduce the level of anxiety and fear in the general population.
- The issue must be discussed individually with each patient and clarification provided on the relationship between ZIKV and microcephaly and other CNS anomalies; women planning to become pregnant must be encouraged to talk to health care providers they trust.
- There is no formal recommendation to avoid pregnancy; the decision of getting pregnant belongs to each woman and her family.
- Eliminating the vector mosquito (Aedes) and preventing mosquito bites are the cornerstones of the battle against ZIKV infection.
- According to Anvisa, women choosing to become pregnant are free to use repellants (Ministério da Saúde - Brazil, 2015).
- Women of advanced reproductive age who wish to postpone pregnancy due to the ZIKV outbreak may have their eggs or embryos frozen and have a chance of enjoying their pregnancies in the future.
- Women suspected for the disease must be counseled to avoid becoming pregnant for at least two months, by natural or assisted reproduction technologies means; infected men must avoid procreation for at least six months from the onset of symptoms.
- Since 80% of infected cases are asymptomatic, and negative serology prior to conception, either natural or through assisted reproduction technologies, does not preclude infection during ovarian stimulation or pregnancy, pre-treatment ZIKV screening may be deemed as an expensive and ineffective measure with low effect.
